# *S*-1-propenylcysteine improves TNF-α-induced vascular endothelial barrier dysfunction by suppressing the GEF-H1/RhoA/Rac pathway

**DOI:** 10.1186/s12964-020-00692-w

**Published:** 2021-02-15

**Authors:** Kayo Kunimura, Satomi Miki, Miyuki Takashima, Jun-ichiro Suzuki

**Affiliations:** Central Research Laboratory, Wakunaga Pharmaceutical Co., Ltd., 624 Shimokotachi, Koda-cho, Akitakata-shi, Hiroshima, 739-1195 Japan

**Keywords:** Vascular endothelial barrier function, Adherens junction, Tight junction, Actin remodeling, *S*-1-propenylcysteine, Aged garlic extract, TNF-α, GEF-H1, RhoA, Rac

## Abstract

**Background:**

Vascular endothelial barrier function is maintained by cell-to-cell junctional proteins and contributes to vascular homeostasis. Various risk factors such as inflammation disrupt barrier function through down-regulation of these proteins and promote vascular diseases such as atherosclerosis. Previous studies have demonstrated that aged garlic extract (AGE) and its sulfur-containing constituents exert the protective effects against several vascular diseases such as atherosclerosis. In this study, we examined whether AGE and its sulfur-containing constituents improve the endothelial barrier dysfunction elicited by a pro-inflammatory cytokine, Tumor-necrosis factor-α (TNF-α), and explored their mode of action on TNF-α signaling pathway.

**Methods:**

Human umbilical vein endothelial cells (HUVECs) were treated with test substances in the presence of TNF-α for various time periods. The endothelial permeability was measured by using a transwell permeability assay. The localization of cell-to-cell junctional proteins and actin cytoskeletons were visualized by immunostaining. RhoA and Rac activities were assessed by using GTP-binding protein pulldown assay. Gene and protein expression levels of signaling molecules were analyzed by real-time PCR and western blotting, respectively.

**Results:**

We found that AGE and its major sulfur-containing constituent, *S*-1-propenylcysteine (S1PC), reduced hyperpermeability elicited by TNF-α in HUVECs. In addition, S1PC inhibited TNF-α-induced production of myosin light chain (MLC) kinase and inactivation of MLC phosphatase through the suppression of the Rac and RhoA signaling pathways, respectively, which resulted in the dephosphorylation of MLC2, a key factor of actin remodeling. Moreover, S1PC inhibited the phosphorylation and activation of guanine nucleotide exchange factor-H1 (GEF-H1), a common upstream key molecule and activator of Rac and RhoA. These effects of S1PC were accompanied by its ability to prevent the disruption of junctional proteins on the cell–cell contact regions and the increase of actin stress fibers induced by TNF-α.

**Conclusions:**

The present study suggested that AGE and its major constituent, S1PC, improve endothelial barrier disruption through the protection of junctional proteins on plasma membrane.
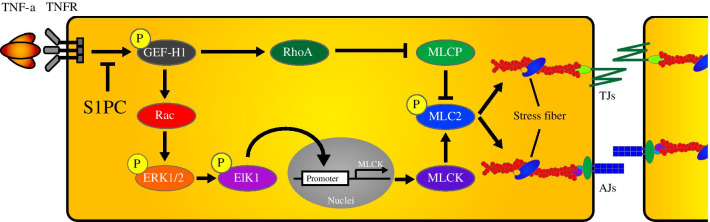

**Video abstract**

## Background

Vascular endothelial cells maintain vascular homeostasis by exerting a barrier function on vascular walls, preventing the infiltration of circulating cells and proteins in blood from vascular lumen into tissues [1, 2]. Chronic inflammation even at low-grade induces disruption of barrier function and enhanced hyperpermeability of vascular endothelium, which leads to the breakdown of vascular homeostasis [2, 3]. This dyshomeostasis causes the invasion of macrophages and neutrophils, inflammation within the artery wall and thereby promotes several vascular diseases including atherosclerosis [4, 5].

Cell–cell junctions play an important role in the maintenance of endothelial barrier function and are organized by tight junctions (TJs), adherens junctions (AJs) and desmosomes in cell–cell contact regions [5]. Among TJ proteins such as Claudins, Occludin and Zona occludens (ZO) proteins, Claudin-5 and Occludin are the major integral transmembrane proteins and form homotypic complexes in endothelial TJs [4–6]. Vascular endothelial cadherin (VE-cadherin), a composition of AJs, also regulates vascular permeability. In addition, the actin of endothelial cells is important for maintaining the cell–cell junctions through the interaction with TJ and AJ proteins. For instance, Claudin-5 and Occludin bind to actin filaments via scaffold proteins ZO-1, ZO-2 and ZO-3 [5, 7]. VE-cadherin also interacts with actin cytoskeleton via p120, β-catenin and several actin-binding proteins [8, 9]. In this way, endothelial barrier function is maintained by controlling these junctional proteins and actin remodeling.

Endothelial barrier dysfunction triggered by inflammation is the first step of various vascular diseases. Tumor necrosis factor-α (TNF-α) is known to disrupt endothelial cell–cell junctions and increase vascular permeability [3, 10]. The failure of vascular integrity elicited by TNF-α is mediated through both TNF receptor type 1 (TNFR1) and TNFR2 signaling pathways [11–13]. TNF-α induces the interaction of myosin and actin stress fibers via enhancing the phosphorylation of myosin light chain (MLC) [14, 15], which subsequently leads to the endocytosis of junctional proteins [16] and thereby causes vascular hyperpermeability. The phosphorylation of MLC is regulated by the balance between MLC kinase (MLCK) and MLC phosphatase (MLCP) activities [17, 18]. After TNF-α stimulation, both RhoA and Rac interact with guanine nucleotide exchange factor (GEF) to activate their downstream molecules. TNF-α inactivates MLCP and activates MLCK, respectively, by the phosphorylation of MLCP through the activation of RhoA/ROCK signaling [15, 18] and by the phosphorylation of MLCK through the activation of Rac signaling [19]. Therefore, the regulation of GEF-mediated activation of Rho and Rac in the TNF-α signaling network is important for the control of vascular permeability.

Aged garlic extract (AGE) is a unique garlic preparation that is produced by aging of raw garlic in an aqueous ethanol solution for more than 10 months. AGE has been shown to exert various biological and pharmacological activities such as anti-inflammatory [20, 21], anti-atherosclerotic [20, 22–24], anti-hypertensive [25, 26] and anti-oxidative effects [27, 28]. In particular, AGE prevented progression of cardiovascular diseases through the improvement of vascular endothelial dysfunction in clinical studies [24, 26, 29–32]. In addition, AGE has been demonstrated to inhibit the lipid deposition [33] and inflammation in a mouse atherosclerosis model [20] and to protect endothelial cells against oxidative injury [34]. AGE contains various sulfur-containing constituents, such as *S*-1-propenylcysteine (S1PC), *S*-allylcysteine (SAC) and *S*-allylmercaptocysteine (SAMC) [35, 36], which are considered to be responsible for some of the pharmacological effects of AGE. S1PC has been shown to inhibit lipopolysaccharide-dependent inflammatory response by activating autophagy and improve hypertension in spontaneously hypertensive rats [35, 37, 38]. SAC and SAMC have been reported to protect endothelial cells by activating antioxidative transcription factor NF-E2-related factor 2 and endothelial NO synthase [27, 39, 40]. Thus, these previous findings suggested that AGE and its sulfur-containing constituents improve endothelial dysfunction.

In the present study, we examined whether AGE and its sulfur-containing constituents affect the barrier function of human vascular endothelial cells. We found that S1PC, a major sulfur-containing constituent of AGE, improved TNF-α-induced endothelial barrier dysfunction by inhibiting both RhoA and Rac pathways through the suppression of the common upstream molecule, GEF-H1, in the TNF-α signaling.

## Materials and methods

### Preparation of AGE and sulfur compounds in AGE

AGE was prepared according to the previous report [38]. Briefly, raw garlic (*Allium sativum* L.) was sliced into pieces, which was followed by immersing in ethanol 20–50% (v/v) and aging for more than 10 months at room temperature. S1PC, SAC and SAMC were synthesized according to the previous methods [41, 42], and the purity was > 99.0%.

### Antibodies and reagents

The following antibodies were used in this study: anti-TNFR1, anti-phospho-GEF-H1 (Ser^886^), anti-GEF-H1, anti-RhoA, anti-phospho-Cofilin (Ser^3^), anti-Cofilin, anti-phospho-MYPT1 (Thr^696^), anti-MYPT1, anti-phospho-MLC2 (Ser^19^), anti-MLC2, horseradish peroxidase (HRP)-conjugated rabbit IgG, HRP-conjugated mouse IgG and HRP-conjugated rat IgG from Cell Signaling Technology (Danvers, MA, USA); anti-VE-cadherin, anti-ZO-1 and Donkey anti-rabbit IgG conjugated to Alexa Fluor 594 from Thermo Fisher Scientific (Waltham, MA, USA); anti-Rac1/2/3, anti-phospho-ETS domain-containing transcription factor 1 (Elk1) (Ser^383^) and anti-Elk1 from Santa cruz Biotechnology (Dallas, TX, USA); anti-TNFR2 and anti-TNFR-associated factor 2 (TRAF2) from Funakoshi (Tokyo, Japan); anti-phospho-ERK1/2 (Thr^202^/Tyr^204^) and anti-ERK1/2 from BioLegend (San Diego, CA, USA); anti-GEF-H1 and anti-β-actin peroxidase conjugate from MBL life science (Nagoya, Japan); anti-Claudin-5 from Bioworld Technology (Nanjing, China); anti-MLCK from Abcam (Cambridge, UK); anti-Glyceraldehyde 3-phosphate dehydrogenase (GAPDH) peroxidase conjugate from WAKO pure chemical industries (Osaka, Japan); IgG from rabbit serum from Merck Millipore (Billerica, MA, USA); acti-stain 488 phalloidin from Cytoskeleton (Denver, CO, USA). Human recombinant TNF-α and human plasma-derived thrombin were purchased from WAKO pure chemical industries and Sigma Aldrich (St. Louis, MO, USA), respectively. Y-27632 and PD98059 were obtained from Merck Millipore and Thermo Fisher Scientific, respectively.

### Cell culture

Human umbilical vein endothelial cells (HUVECs) were purchased from Lonza (Basel, Switzerland) and grown to be confluent in endothelial cell basal medium-2 (Lonza) containing 2% fetal bovine serum albumin (BSA) and supplements attached to the medium kit in the presence of 5% CO_2_ at 37 °C.

### In vitro endothelial permeability assay

Endothelial permeability was measured by using in vitro vascular permeability assay kit (Merck Millipore). HUVECs were seeded at 1.5 × 10^3^ cells/well on transwell inserts and cultured in a receiver plate. After grown to be confluent, HUVECs were stimulated with 50 ng/mL TNF-α or 1 U/mL thrombin in the absence or presence of test substances (1–4 mg/mL AGE, 75–300 μM S1PC, 300 μM SAC or 300 μM SAMC) for 24 h. In the case of the study using the inhibitors of RhoA and Rac signaling molecules, HUVECs were pre-treated with Y-27632 (a ROCK inhibitor, 10 μM) or PD98059 (a MEK1 inhibitor, 30 μM) for 1 h before the TNF-α stimulation. Then, FITC-dextran was added on each transwell insert and the extent of permeability was determined by measuring the fluorescence of each receiver plate solution. The fluorescence intensity was quantified with EnVision plate reader (PerkinElmer, Waltham, MA, USA) at an excitation wavelength of 485 nm and an emission wavelength of 535 nm.

### Western blot analysis

After seeding on 24-well plates, HUVECs were stimulated with TNF-α 50 ng/mL in the absence or presence of test substances (75–300 μM S1PC, 300 μM SAC or 300 μM SAMC) for 10, 15, 20, 30, 40 min, 1, 3 or 24 h. In the case of the study using the inhibitors of RhoA and Rac signaling molecules, HUVECs were pre-treated with 10 μM Y-27632 or 30 μM PD98059 for 1 h before the TNF-α stimulation. Then, whole protein was extracted using Radioimmunoprecipitation assay (RIPA) buffer (WAKO pure chemical industries) containing protease and phosphatase inhibitor cocktail (Thermo Fisher Scientific). After dilution with SDS sample buffer (62.5 mM Tris-HCl (pH 6.8), 2% SDS, 10% glycerol, 0.005% bromophenol blue, 175 mM dithiothreitol), 10 μg of total protein was separated by SDS-PAGE and transferred onto nitrocellulose membrane (Bio-Rad Laboratories, Hercules, CA, USA). The transferred proteins were incubated with antibodies and immunoreactive proteins were detected with ECL prime western blotting detection system (GE Healthcare, Little Chalfont, Buckinghamshire, UK) or Supersignal west femto maximum sensitivity substrate (Thermo Fisher Scientific). Chemiluminescent signals were visualized with ChemiDoc MP imaging system (Bio-Rad Laboratories) and quantified using ImageLab software (Bio-Rad Laboratories).

### Extraction of membrane and cytoplasmic proteins

HUVECs were seeded on 12-well plates and stimulated with TNF-α 50 ng/mL in the absence or presence of 300 μM S1PC for 30 min or 24 h. Then, cell membrane and cytoplasmic proteins were separately isolated by using Mem-PER™ plus membrane protein extraction kit (Thermo Fisher Scientific) according to the manufacturer’s protocol. The isolated membrane and cytoplasmic proteins were analyzed by western blotting. The total proteins were stained by using Quick-coomassie brilliant blue staining kit (WAKO pure chemical industries) according to the manufacturer’s instructions in order to normalize the membrane and cytoplasmic protein levels.

### Immunoprecipitation

Immunoprecipitation was performed to investigate the interaction between GEF-H1 and Rho family proteins. HUVECs were seeded on 10 cm dishes and stimulated with TNF-α 50 ng/mL in the absence or presence of 300 μM S1PC for 1 h. Then, whole proteins were isolated using RIPA buffer containing protease and phosphatase inhibitor cocktail. Cell lysates were immunoprecipitated using GEF-H1 antibody and protein G magnetic beads (Merck Millipore). The magnetic beads were washed 5 times by RIPA buffer and boiled in SDS sample buffer at 95 °C for 5 min. Finally, the released proteins from beads were analyzed by western blotting.

### Immunofluorescent staining

To investigate the effect of S1PC on cellular localization of the junctional proteins and F-actin, these proteins were visualized by immunofluorescent staining. HUVECs were seeded on 8-well slide chambers (WATSON, Tokyo, Japan) and stimulated with TNF-α 50 ng/mL in the absence or presence of 300 μM S1PC for 24 h. Then, the cells were fixed in 4% paraformaldehyde (WAKO pure chemical industries), permeabilizated with 0.3% TritonX-100 (Sigma Aldrich) in phosphate buffered saline (PBS, NISSUI, Tokyo, Japan) and blocked with 3% BSA (Sigma Aldrich) in PBS followed by the incubation with primary antibodies in 3% BSA/PBS. Then, the cells were treated with an Alexa Fluor 594-conjugated secondary antibody to detect junctional proteins. F-actin was labeled by the incubation with acti-stain 488 phalloidin. Finally, the cells were mounted using Vectashield medium-containing 4′,6-diamidino-2-phenylindole (DAPI) (Vector Laboratories, Berlingame, CA, USA). The fluorescent staining was visualized with Biorevo Bz9000 fluorescence microscope (Keyence, Osaka, Japan) and analyzed using Bz-II analyzer software (Keyence).

### RhoA and Rac activation assay

The effects of S1PC on the activities of Rho family proteins were evaluated by using RhoA and Rac activation assay kits (Merck Millipore). HUVECs were seeded on 10 cm dishes and stimulated with TNF-α 50 ng/mL in the absence or presence of 300 μM S1PC for 1 h. After being detached using Mg^2+^ lysis/wash buffer (MLB) containing protease and phosphatase inhibitor cocktail, the cells were incubated with glutathione-agarose beads (Sigma Aldrich) for 30 min at 4 °C. Then, the supernatant collected from the incubation medium was added with Rho or Rac assay reagent containing glutathione-agarose beads bound to fusion proteins that include Rho or Rac binding domain of each effector protein, and then gently agitated. After centrifugation, the precipitated bead pellets were washed 3 times using MLB and boiled in SDS sample buffer. Finally, the supernatants were analyzed by western blotting.

### Quantitative real-time PCR

HUVECs were seeded on 48-well plates and stimulated with TNF-α 50 ng/mL in the absence or presence of 300 μM S1PC for 9 h. Then, total RNA was isolated using TRIzol reagent (Thermo Fisher Scientific) and used for the synthesis of the first-strand cDNA by using Primescript RT reagent kit with gDNA Eraser (Takara, Shiga, Japan). The quantitative real-time PCR was performed with Piko Real Real-time PCR system (Life technologies, Carlsbad, CA, USA) using SYBR premix Ex Taq II (Takara). The primer sequences used for PCR were as follows: human *MLCK* forward, 5ʹ-AGCCCGCTCAATGCAGAAAA-3ʹ; human *MLCK* reverse, 5ʹ-AGCAGCACTTCCCTCCACAA-3ʹ; human *β-Actin* forward, 5ʹ-CGCGAGAAGATGACCCAGAT-3ʹ; human *β-Actin* reverse, 5ʹ-GGTGAGGATCTTCATGAGGTAGTC-3ʹ. The relative mRNA expression level of MLCK to β-Actin was calculated using the comparative CT (ΔΔCT) method.

### Knockdown of GEF-H1 gene with small interfering RNA (siRNA)

To examine whether GEF-H1 is a key molecule of TNF-α-activated RhoA and Rac signaling pathways, we prepared GEF-H1 knockdown HUVECs with siRNA. HUVECs seeded on 12-well plates were transfected with 9.6 pmol control siRNA (Silencer select negative control No. 1 siRNA, Thermo Fisher Scientific) or GEF-H1-targeting siRNA (Silencer select pre-designed hARFGEF2 siRNA, ID: s17546, Thermo Fisher Scientific) using Lipofectamine RNAiMAX reagent (Thermo Fisher Scientific) according to the manufacturer’s instructions. At 48 h after the transfection, the cells were stimulated with TNF-α 50 ng/mL for 15, 30 min, 1 or 24 h, and the levels of protein expressions were evaluated by western blotting.

### Cell proliferation assay

Cell proliferation assay was performed to investigate the effect of S1PC on the proliferation of HUVECs by using Cell counting kit-8 (Dojindo Molecular Technologies, Kumamoto, Japan). Briefly, HUVECs seeded on 96-well plates were treated with 75–300 μM S1PC for 48 or 72 h, and each well of the plates was added with CCK-8 regent and incubated for 1 h. The colorimetric absorbance of the wells was measured at 450 nm with Multiskan FC microplate reader (Thermo Fisher Scientific).

### Statistical analysis

Data were expressed as means ± standard deviation (SD) after outlier data were detected and removed using Thompson's rejection test. Bonferroni’s or Dunnett’s multiple comparison test was performed to assess statistically significant differences between groups by using WinSTAT statistics software (M. Sato, Japan) or KyPlot software (KyensLab, Tokyo, Japan), respectively. Differences at *P* values less than 0.05 were considered to be statistically significant.

## Results

### S1PC suppressed endothelial hyperpermeability by improving the expression of junctional proteins

**Fig. 1:**
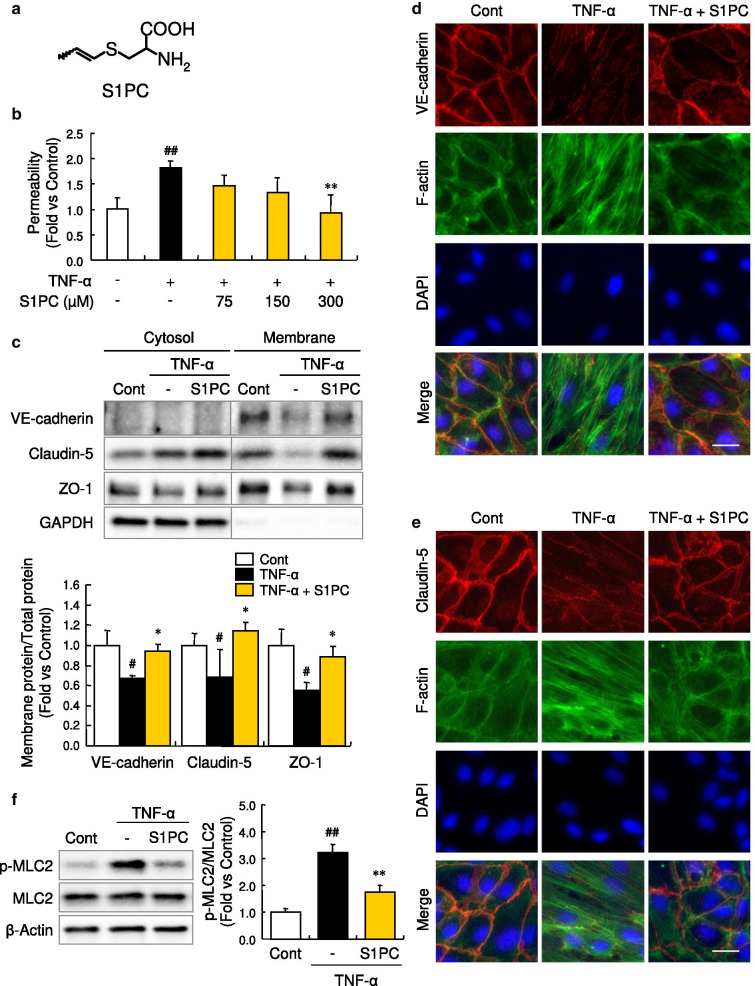
Effects of S1PC on cell permeability, junctional/cytoskeletal proteins, and MLC2 phosphorylation in TNF-α-stimulated HUVECs. **a** Chemical structure of S1PC. **b** The concentration-dependent effect of S1PC on TNF-α-induced hyperpermeability of HUVECs. Vascular permeability assay was conducted after the stimulation with TNF-α (50 ng/mL) in the presence or absence of S1PC (75, 150 or 300 μM) for 24 h. Data are shown as mean ± SD, n = 4. Significant difference compared to the control group (^##^*p* < 0.01) or TNF-α-treated group (**p < 0.01) was determined by Dunnett’s multiple comparison test. **c** Effect of S1PC on TNF-α-induced disruption of junctional proteins in HUVECs. The membrane and cytoplasmic proteins were extracted after the stimulation with TNF-α (50 ng/mL) in the presence or absence of S1PC (300 μM) for 24 h and analyzed by western blotting with indicated antibodies. Quantitative data are shown as mean ± SD, n = 3–6. Significant difference compared to the control group (^#^*p* < 0.05) or TNF-α-treated group (*p < 0.05) was determined by Bonferroni’s multiple comparison test. **d**, **e** Effect of S1PC on TNF-α-induced disturbance of localization of junctional proteins, VE-cadherin and Claudin-5, and actin cytoskeleton in HUVECs. HUVECs were immunostained with indicated antibodies after the stimulation with TNF-α (50 ng/mL) in the presence or absence of S1PC (300 μM) for 24 h. Specific fluorescence: red for VE-cadherin (**d**) and Claudin-5 (**e**), green for F-actin and blue for nuclei stained with DAPI. Scale bar, 25 μm. **f** Effect of S1PC on TNF-α-induced MLC2 phosphorylation in HUVECs. Cell lysates were obtained after the stimulation with TNF-α (50 ng/mL) in the presence or absence of S1PC (300 μM) for 24 h and analyzed by western blotting with indicated antibodies. Quantitative data are shown as mean ± SD, n = 3. Significant difference compared to the control group (^##^*p* < 0.01) or TNF-α-treated group (**p < 0.01) was determined by Bonferroni’s comparison test

We first evaluated the effect of AGE on endothelial permeability of HUVECs by using a transwell permeability assay. The permeability was evaluated by fluorescently measuring the transport of FITC-dextran from transwell inserts to the receiver plate wells through the monolayered HUVECs. Stimulation with TNF-α 50 ng/mL for 24 h increased cellular permeability in HUVECs. AGE prevented the increase in the transport of FITC-dextran induced by TNF-α in a concentration-dependent manner (Additional file 1: Figure S1a). To identify the responsible constituents of AGE, we next examined the protective effects of three sulfur-containing constituents, S1PC (Fig. 1a), SAC and SAMC (Additional file 1: Figure S1b), against the endothelial permeability in HUVECs. As shown in Fig. 1b, S1PC significantly inhibited the TNF-α-induced transport of FITC-dextran at the concentration of 300 μM, while the other two constituents did not (Additional file 1: Figure. S1c). In addition, S1PC inhibited endothelial hyperpermeability induced by 1 U/mL thrombin (Additional file 2: Figure S2), that is a serine protease well known for its endothelial hyperpermeability effect [43, 44]. Since the disruption of AJ and TJ proteins causes vascular hyperpermeability [1, 9, 10, 45], we investigated the effect of S1PC on the amount of these proteins in the membrane fraction of HUVECs. As shown in Fig. 1c, the protein levels of two transmembrane proteins, VE-cadherin and Claudin-5, in the plasma membrane fraction were decreased after the treatment with TNF-α 50 ng/mL, but co-treatment with S1PC restored the reduced protein levels to the control level (vehicle treatment). In addition, S1PC also restored the level of scaffold protein ZO-1 in the plasma membrane fraction. Furthermore, immunofluorescent staining also revealed that S1PC suppressed the TNF-α-induced down-regulation of VE-cadherin and Claudin-5 at the cellular membranes (Fig. 1d, e).

We next evaluated the effect of S1PC on actin remodeling since TNF-α has been reported to induce the degradation of junctional proteins via the overproduction of actin filaments [16]. As shown in Fig. 1d, e, treatment with TNF-α increased actin stress fibers in HUVECs, whereas co-treatment with S1PC inhibited TNF-α-induced actin polymerization. The phosphorylation of MLC2 elicited by TNF-α has been reported to trigger actin polymerization and generate actomyosin contractile force [14, 15]. As shown in Fig. 1f, MLC2 phosphorylation was enhanced by the treatment with TNF-α for 24 h that was also inhibited by co-treatment with S1PC. Taken together, these results suggested that S1PC prevented endothelial barrier dysfunction induced by TNF-α through the suppression of MLC2-inudced down-regulation of junctional proteins in the TNF-α signaling.

### S1PC inhibited GEF-H1 phosphorylation induced by TNF-α

To reveal the underlying mechanism of the inhibitory effect of S1PC on TNF-α-induced MLC2 phosphorylation, we examined the effects of S1PC on the expression of TNFR and its adaptor protein TRAF2. S1PC, however, had no effect on these protein levels in the plasma membrane fraction of HUVECs (Additional file 3: Figures S3a and S3b), suggesting that S1PC may act on the downstream molecules of the TNF-α signaling pathway.

**Fig. 2:**
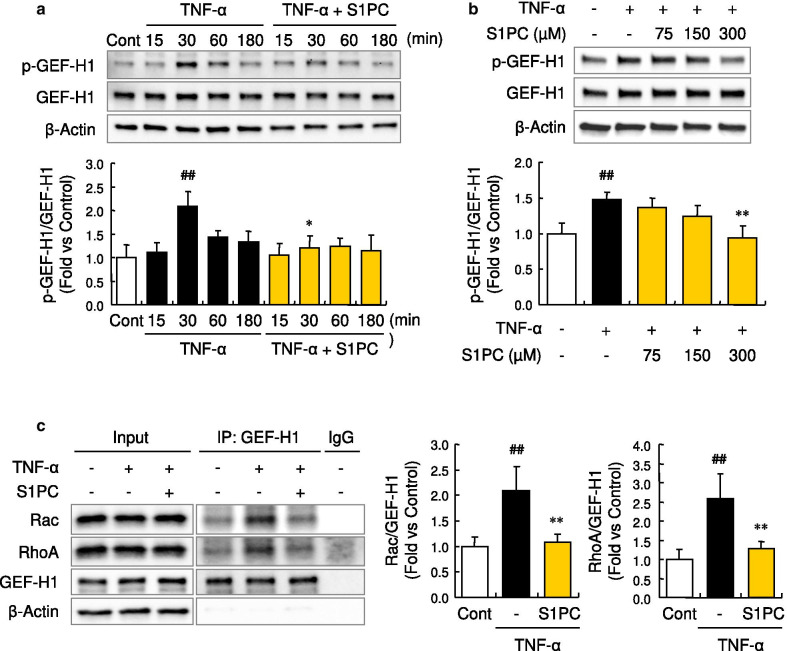
Effects of S1PC on TNF-α-induced phosphorylation and interaction with Rho family of GEF-H1 in HUVECs. **a** Effect of S1PC on TNF-α-induced GEF-H1 phosphorylation in HUVECs. Cell lysates were obtained after the stimulation with TNF-α (50 ng/mL) in the presence or absence of S1PC (300 μM) for 0.25, 0.5, 1 or 3 h and analyzed by western blotting with indicated antibodies. Quantitative data are shown as mean ± SD, n = 3. Significant difference compared to the control group (^##^*p* < 0.01) or TNF-α-treated group with the same treatment time (*p < 0.05) was determined by Bonferroni’s multiple comparison test. **b** The concentration-dependent effect of S1PC on TNF-α-induced GEF-H1 phosphorylation in HUVECs. Cell lysates were obtained after the stimulation with TNF-α (50 ng/mL) in the presence or absence of S1PC (75, 150 or 300 μM) for 30 min and analyzed by western blotting with indicated antibodies. Quantitative data are shown as mean ± SD, n = 4. Significant difference compared to the control group (^##^*p* < 0.01) or TNF-α-treated group (**p < 0.01) was determined by Dunnett’s multiple comparison test. **c** Effect of S1PC on TNF-α-induced interactions of GEF-H1 with Rho family proteins, RhoA (right graph) and Rac (left graph) in HUVECs. Cell lysates were obtained after the stimulation with TNF-α (50 ng/mL) in the presence or absence of S1PC (300 μM) for 1 h and immunoprecipitated with anti-GEF-H1 antibody (IP: GEF-H1) or control antibody (IgG). Then the precipitated proteins and total proteins (Input) were analyzed by western blotting with indicated antibodies. Quantitative data are shown as mean ± SD, n = 3–4. Significant difference compared to the control group (^##^*p* < 0.01) or TNF-α-treated group (**p < 0.01) was determined by Bonferroni’s multiple comparison test

GEF-H1, one of the downstream molecules activated by TNF-α, has been known to activate both RhoA and Rac. As shown in Fig. 2a, b, S1PC inhibited the phosphorylation of GEF-H1 induced by the treatment with TNF-α in a concentration-dependent manner. On the other hand, SAC and SAMC showed no inhibitory effect on the GEF-H1 phosphorylation as in the case of the permeability (Additional file 1: Figure S1c and Additional file 4: Figure S4). Furthermore, S1PC blocked the formation of GEF-H1/RhoA/Rac complex, the downstream event of GEF-H1 activation in TNF-α signaling pathway (Fig. 2c). Since GEF-H1 is also reported to be associated with G1/S phase transition and cytokinesis of cells [46, 47], the severe inhibition of GEF-H1 and its downstream signals could affect cell proliferation. S1PC inhibited TNF-α-induced phosphorylation of GEF-H1 but had no effect on the cell growth and cytokinesis (Additional file 5: Figures S5a and S5b).

### S1PC inhibited RhoA activation and its downstream signaling induced by TNF-α

**Fig. 3:**
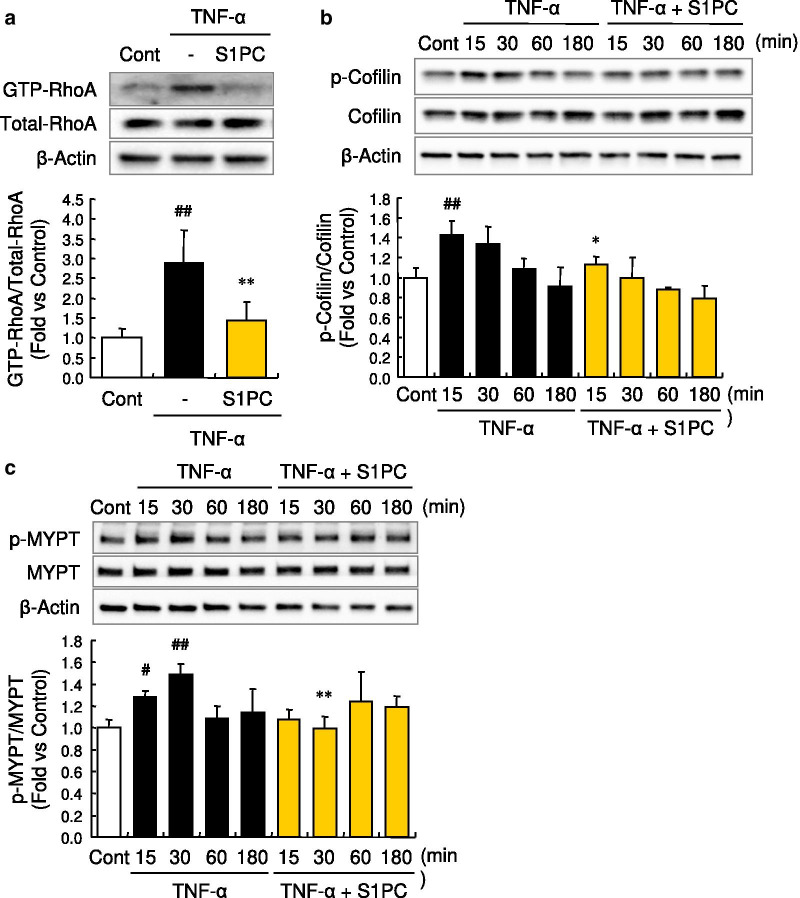
Effect of S1PC on a downstream signaling of TNF-α, RhoA, in HUVECs. **a** Effect of S1PC on TNF-α-induced RhoA activity in HUVECs. The active GTP-bound RhoA (GTP-RhoA) was obtained with RhoA activation assay system after the stimulation with TNF-α (50 ng/mL) in the presence or absence of S1PC (300 μM) for 1 h and analyzed by western blotting with indicated antibodies. Quantitative data are shown as mean ± SD, n = 3–6. Significant difference compared to the control group (^##^*p* < 0.01) or TNF-α-treated group (**p < 0.01) was determined by Bonferroni’s comparison test. **b**,** c** Effects of S1PC on TNF-α-induced phosphorylation of Cofilin (**b**) and MYPT (**c**) in HUVECs. Cell lysates were obtained after the stimulation with TNF-α (50 ng/mL) in the presence or absence of S1PC (300 μM) for 0.25, 0.5, 1 or 3 h and analyzed by western blotting with indicated antibodies. Quantitative data are shown as mean ± SD, n = 3. Significant difference compared to the control group (^##^*p* < 0.01, ^#^*p* < 0.05) or TNF-α-treated group with the same treatment time (**p < 0.01, *p < 0.05) was determined by Bonferroni’s multiple comparison test

We next investigated whether the inhibitory effect of S1PC on the GEF-H1 phosphorylation affects the RhoA signaling. In this signaling pathway, RhoA activates ROCK that phosphorylates both an actin-binding protein cofilin and MLCP regulatory subunit (MYPT) at Ser^3^ and Thr^696^, respectively. The phosphorylated cofilin abolishes the disassembly activity of actin filament and the phosphorylated MYPT negatively regulates MLCP activity resulting in enhanced MLC2 phosphorylation, which in turn leads to actin polymerization and actin-myosin contraction [46, 48, 49]. First, we confirmed that GEF-H1 is a key molecule in the TNF-α-activated RhoA signaling pathway since the knockdown of GEF-H1 by its siRNA suppressed the downstream signaling of RhoA, the phosphorylations of MLC2 and MYPT, induced by TNF-α in HUVECs (Additional file 6: Figures S6a and S6b). We then evaluated the effect of S1PC on the RhoA signaling. As shown in Fig. 3a, co-treatment of HUVECs with S1PC suppressed the increased level of the activated form of RhoA, its GTP-bound form, induced by the treatment with TNF-α. In addition, S1PC inhibited the phosphorylation of both cofilin and MYPT induced by TNF-α (Fig. 3b, c). Taken together, these data suggested that GEF-H1 plays an important role in TNF-α-induced MYPT phosphorylation and S1PC suppresses the RhoA signaling pathway activated by TNF-α via acting on GEF-H1.

### S1PC inhibited Rac activation and its downstream signaling induced by TNF-α

**Fig. 4:**
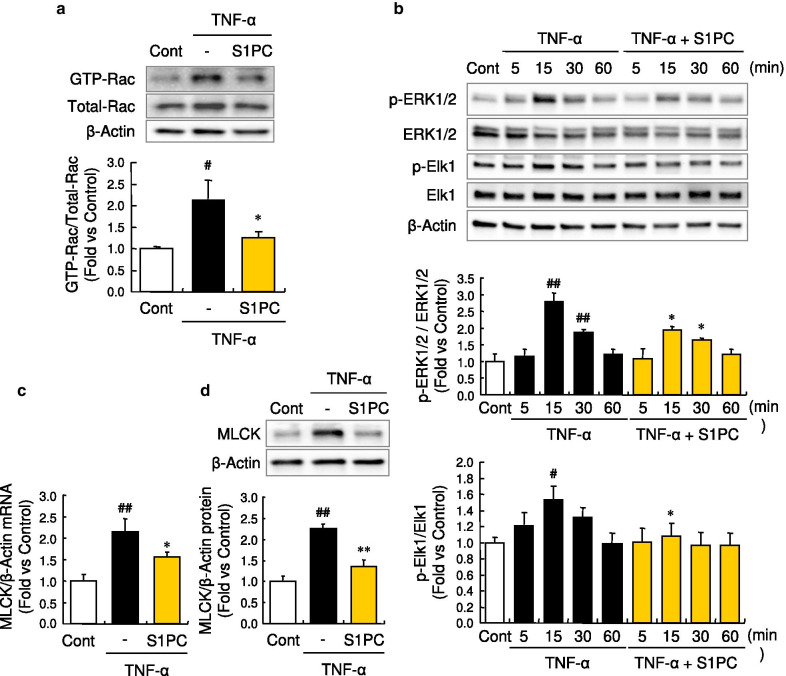
Effect of S1PC on a downstream signaling of TNF-α, Rac, in HUVECs. **a** Effect of S1PC on TNF-α-induced Rac activity in HUVECs. The active GTP-bound Rac (GTP-Rac) was obtained with Rac activation assay system after the stimulation with TNF-α (50 ng/mL) in the presence or absence of S1PC (300 μM) for 1 h and analyzed by western blotting with indicated antibodies. Quantitative data are shown as mean ± SD, n = 3. Significant difference compared to the control group (^#^*p* < 0.05) or TNF-α-treated group (*p < 0.05) was determined by Bonferroni’s comparison test. **b** Effects of S1PC on TNF-α-induced phosphorylation of ERK1/2 (upper graph) and Elk1 (lower graph) in HUVECs. Cell lysates were obtained after the stimulation with TNF-α (50 ng/mL) in the presence or absence of S1PC (300 μM) for 5, 15, 30 or 60 min and analyzed by western blotting with indicated antibodies. Quantitative data are shown as mean ± SD, n = 3. Significant difference compared to the control group (^##^*p* < 0.01, ^#^*p* < 0.05) or TNF-α-treated group with the same treatment time (*p < 0.05) was determined by Bonferroni’s multiple comparison test. **c**, **d** Effects of S1PC on TNF-α-induced gene (**c**) and protein (**d**) expression of MLCK in HUVECs. Total mRNA or cell lysates were obtained after the stimulation with TNF-α (50 ng/mL) in the presence or absence of S1PC (300 μM) for 9 or 24 h and analyzed by quantitative real-time PCR analysis or western blotting with indicated antibodies, respectively. Data are shown as mean ± SD, n = 3–4. Significant difference compared to the control group (^##^*p* < 0.01) or TNF-α-treated group (**p < 0.01, *p < 0.05) was determined by Bonferroni’s comparison test

We investigated whether the inhibitory effect of S1PC on the GEF-H1 phosphorylation affects the Rac signaling. In this signaling pathway, Rac promotes ERK phosphorylation by activating MEK, and then ERK phosphorylates Elk1 at Ser^383^ [50]. Following the nuclear translocation, phosphorylated Elk1 binds to the promoter region and induces gene transcription of MLCK [51]. We first confirmed that GEF-H1 is a key molecule of TNF-α-activated Rac signaling pathway since the knockdown of GEF-H1 by its siRNA suppressed the downstream signaling of Rac, ERK1/2 phosphorylation and protein expression of MLCK, induced by TNF-α in HUVECs (Additional file 6: Figures S6c and S6d). We next assessed the effect of S1PC on the Rac signaling pathway. As shown in Fig. 4a, co-treatment of HUVECs with S1PC suppressed the increased level of activated form of Rac, its GTP-bound form, induced by the treatment with TNF-α. In addition, S1PC significantly inhibited the phosphorylations of ERK1/2 and Elk1 as well as the increased mRNA and protein levels of MLCK induced by TNF-α (Fig. 4b–d). Altogether, these results indicated that GEF-H1 plays an important role in TNF-α-induced MLCK production and S1PC inhibits the Rac signaling pathway activated by TNF-α via acting on GEF-H1.

### Both inhibition of RhoA and Rac signaling pathways were required to restore the vascular endothelial dysfunction induced by TNF-α

We investigated the effects of Y-27632, a ROCK inhibitor, and PD98059, a MEK1 inhibitor, on the RhoA and Rac signaling pathways, respectively, and vascular permeability in HUVECs. Y-27632 reduced the phosphorylation of MYPT but not the phosphorylation of ERK1/2 induced by TNF-α (Additional file 7: Figure S7). On the other hand, PD98059 reduced the production of MLCK but not the phosphorylation of MYPT induced by TNF-α (Additional file 8: Figures S8a and S8b). Neither Y-27632 nor PD98059 significantly restored the vascular permeability impaired by TNF-α (Additional file 9: Figures S9a and S9b). These data indicated that RhoA/ROCK and Rac/ERK1/2 pathways independently act as the downstream signaling of GEF-H1 in the TNF-α signaling, and that the inhibition of both RhoA and Rac signaling may be required for the protection against vascular endothelial dysfunction induced by TNF-α.

## Discussion

Vascular endothelial barrier dysfunction is not only a key early event associated with the development of cardiovascular diseases but also is important throughout disease trajectory [52, 53]. It is also strongly implicated in a variety of disease states. The leakage of microcirculatory flow caused by increasing endothelial permeability contributes to the progression of life-threatening illnesses such as sepsis [54, 55], stroke [56], acute respiratory distress syndrome [57] and others, subsequently inducing organ failure [52, 58]. In addition, the disruption of blood–brain barrier tight junction is associated with cognition impairment via central nervous system injury [59, 60]. Thus, maintaining homeostasis of the barrier function in the vascular endothelium is important for protection against a variety of end-organ diseases throughout their development.

The barrier function of vascular endothelium is regulated by cell–cell junctions and the disruption of these assembly contributes to the increased endothelial permeability. In the present study, we found that AGE and its sulfur-containing constituent, S1PC, improved the TNF-α-induced endothelial barrier dysfunction through the regulation of AJs and TJs assembly (Fig. 1 and Additional file 1: Figure S1a). In addition, this compound also restored the thrombin-induced hyperpermeability (Additional file 2: Figure S2). Therefore, S1PC might prevent vascular endothelial hyperpermeability induced by several risk factors.

Previous clinical studies reported that AGE reduced low attenuation plaque in coronary arteries of diabetic patients and inhibited the progression of atherosclerotic lesion [22–24]. Atherosclerotic plaque is formed by the infiltration of monocytes and accumulation of oxidized low-density lipoprotein caused by endothelial barrier dysfunction [61, 62]. These findings suggested that anti-atherosclerotic effect of AGE may be attributable to its ability to protect and maintain the vascular endothelial barrier function.

AGE has been reported to exert various pharmacological effects, such as anti-atherosclerotic [20, 22–24], anti-hypertensive [25, 26] and anti-inflammatory effects [20, 21], which are considered to be attributable to its sulfur-containing constituents. Therefore, we investigated whether S1PC, SAC and SAMC, three major sulfur-containing constituents in AGE, affect the endothelial barrier function. Consequently, we found that S1PC but not SAC and SAMC inhibited the hyperpermeability of HUVECs induced by TNF-α (Fig. 1b and Additional file 1: Figure S1c). These three constituents have similar structures, and especially S1PC and SAC are structural isomers and differ only in the position of the double bond (Fig. 1a and Additional file 1: Figure S1b). It is therefore suggested that the position of double bond is important for the ability of S1PC to maintain the cell–cell junctional assembly.

TNF-α elicits the hyperphosphorylation of MLC2 by inducing the imbalance between MLCK and MLCP activities [17, 18], which subsequently triggers the endocytosis of junctional proteins by remodeling of actin cytoskeleton and eventually leads to the dysfunction of vascular endothelial barrier [16]. Our findings indicated that S1PC protected against TNF-α-induced barrier dysfunction through the MLC2 dephosphorylation via downregulation of MLCK and prevention of MLCP inhibition in the TNF-α signaling pathway (Figs. 1f, 3c, 4c, d), resulting in the maintenance of proper localization of junctional proteins by inhibiting actin polymerization (Fig. 1d, e). The activities of MLCK and MLCP are regulated, respectively, through Rac/ERK1/2 signaling and RhoA/ROCK signaling in the TNF-α downstream pathway [15, 18, 19]. Thus, we investigated which one is a key signaling for the induction of vascular endothelial hyperpermeability. However, the abolishment of either Rac/ERK1/2 or RhoA/ROCK signaling by each selective inhibitor was not sufficient to improve hyperpermeability induced by TNF-α (Additional file 9: Figures S9a and S9b), suggesting that blockade of both Rac/ERK1/2 and RhoA/ROCK signaling is required to inhibit TNF-α-induced hyperpermeability.

RhoA and Rac comprise a family of Rho GTPase and their activities are regulated by various GEFs. GEF-H1 has been known to activate both RhoA and Rac in response to the stimulation of TNF-α and thrombin [46, 63, 64]. Our study demonstrated that S1PC inhibited both RhoA and Rac signaling activated by TNF-α (Figs. 3a, 4a); S1PC inhibited the inactivation of MLCP and increase of MLCK production induced by TNF-α, collectively contributing to the inhibition of MLC2 phosphorylation (Figs. 1f, 3c, 4c, d). GEF-H1, the common upstream molecule of RhoA and Rac signaling pathways, has multiple phosphorylation sites regulating its own activity [64, 65]. TNF-α induced GEF-H1 phosphorylation at Ser^885^ (Ser^886^ in human) in the N-terminal domain that is essential to activate both RhoA and Rac [64, 65]. Our study showed that S1PC decreased the phosphorylation level of GEF-H1 and suppressed the interaction of GEF-H1 with RhoA and Rac (Fig. 2). Taken together, these results showed that S1PC protected against the barrier dysfunction elicited by TNF-α through inhibiting the phosphorylation and activation of GEF-H1, and suggested that this effect of S1PC is associated with the beneficial effect of AGE on atherosclerotic patients observed in the clinical studies [22–24].

Apart from the role in the regulation of endothelial junctions, GEF-H1 is involved in several cellular events such as cell division [47, 66, 67], differentiation [68], motility and polarization [69]. It is likely that the severe inhibition of GEF-H1 also impairs these essential cell events. For instance, it was reported that the depletion of GEF-H1 increased the number of multinuclear cells through induction of cytokinesis failure and reduced the cell growth [47]. However, we confirmed that the inhibition of GEF-H1 by S1PC did not affect cytokinesis and the cell growth (Additional file 5: Figure S5), suggesting that S1PC could improve the vascular endothelial dysfunction without impairing the other essential functions of GEF-H1.

## Conclusion

This study showed that S1PC, a sulfur-containing constituent in AGE, protected against the TNF-α-induced disruption of endothelial junctions in HUVECs by suppressing the GEF-H1 activation in the signaling pathway. Vascular endothelial TJs act as a primary barrier for maintaining the homeostasis and function of blood vessels and organs. Accordingly, the proper maintenance of barrier function by S1PC may help protect the blood vessels against risk factors of vascular and organ diseases. In addition, the protective effect of S1PC on vascular cells may explain in part the beneficial effect of AGE on the atherosclerotic patients in the clinical studies [22–24].

## Supplementary Information

**Additional file 1: Figure S1.** Effects of AGE and its major sulfur-constituents on TNF-α-induced hyperpermeability of HUVECs. **a** The concentration-dependent effect of AGE on TNF-α-induced hyperpermeability of HUVECs. Vascular permeability assay was conducted after the stimulation with TNF-α (50 ng/mL) in the presence or absence of AGE (1, 2 or 4 mg/mL) for 24 h. Data are shown as mean ± SD, n = 3–4. Significant difference compared to the control group (^##^*p* < 0.01) or TNF-α-treated group (**p < 0.01) was determined by Dunnett’s multiple comparison test. **b** Chemical structures of SAC and SAMC. **c** Effects of major sulfur-containing constituents of AGE on TNF-α-induced hyperpermeability of HUVECs. Vascular permeability was conducted after the stimulation with TNF-α (50 ng/mL) in the presence or absence of sulfur-containing constituents of AGE (300 μM S1PC, SAC or SAMC) for 24 h. Data are shown as mean ± SD, n = 4. Significant difference compared to the control group (^#^*p* < 0.05) or TNF-α-treated group (*p < 0.05) was determined by Bonferroni’s multiple comparison test.

**Additional file 2: Figure S2.** The concentration-dependent effect of S1PC on thrombin-induced hyperpermeability of HUVECs. Vascular permeability assay was conducted after the stimulation with thrombin (1 U/mL) in the presence or absence of S1PC (75, 150 or 300 μM) for 24 h. Data are shown as mean ± SD, n = 4. Significant difference compared to the control group (^##^*p* < 0.01) or TNF-α-treated group (**p < 0.01) was determined by Dunnett’s multiple comparison test.

**Additional file 3: Figure S3.** Effects of S1PC on protein levels of TNF receptors and TRAF2 in HUVECs. **a** Effects of S1PC on the protein levels of TNFR1 and TNFR2 in HUVECs. The membrane and cytoplasmic proteins were extracted after the stimulation with TNF-α (50 ng/mL) in the presence or absence of S1PC (300 μM) for 24 h and analyzed by western blotting with indicated antibodies. Quantitative data are shown as mean ± SD, n = 3. **b** Effect of S1PC on the protein level of adaptor protein TRAF2 in HUVECs. The membrane and cytoplasmic proteins were extracted after the stimulation with TNF-α (50 ng/mL) in the presence or absence of S1PC (300 μM) for 30 min and analyzed by western blotting with indicated antibodies. Quantitative data are shown as mean ± SD, n = 4.

**Additional file 4: Figure S4.** Effects of major sulfur-containing constituents of AGE on TNF-α-induced GEF-H1 phosphorylation in HUVECs. Cell lysates were obtained after the stimulation with TNF-α (50 ng/mL) in the presence or absence of sulfur-containing constituents of AGE (300 μM S1PC, SAC and SAMC) for 30 min and analyzed by western blotting with indicated antibodies. Quantitative data are shown as mean ± SD, n = 3. Significant difference compared to the control group (^##^*p* < 0.01) or TNF-α-treated group (*p < 0.05) was determined by Bonferroni’s multiple comparison test.

**Additional file 5: Figure S5.** Effects of S1PC on the cell proliferation and morphology of HUVECs. **a** Effect of S1PC on the cell proliferation in HUVECs. Cell viability was measured with cell proliferation assay system after the treatment with S1PC (75, 150 or 300 μM) for 48 and 72 h. Data are shown as mean ± SD, n = 3. **b** Effect of S1PC on the cell morphology in HUVECs. Cell nuclei were stained with DAPI to assess normal cytokinesis after the treatment with S1PC (300 μM) for 24 h. Specific fluorescence: blue for nuclei stained with DAPI. Scale bar, 100 μm.

**Additional file 6: Figure S6.** Effects of GEF-H1 knockdown on downstream signaling of TNF-α, RhoA and Rac, in HUVECs. Effects of GEF-H1 knockdown on TNF-α-induced MLC2 phosphorylation (**a**), MYPT phosphorylation (**b**), ERK1/2 phosphorylation (**c**) and MLCK protein expression (**d**) were examined in HUVECs. HUVECs were transfected with control siRNA (siControl) or GEF-H1-targeting siRNA (siGEF-H1). Cell lysates were obtained after the stimulation with TNF-α (50 ng/mL) for 24 h (**a**, **d**) or indicated periods (**b**, **c**), and analyzed by western blotting with indicated antibodies. Quantitative data are shown as mean ± SD, n = 3. Significant difference compared to non-treatment siControl group (^##^*p* < 0.01, ^#^*p* < 0.05) or TNF-α-treated siControl group with the same treatment time (**p < 0.01, *p < 0.05) was determined by Bonferroni’s multiple comparison test.

**Additional file 7: Figure S7.** Effects of ROCK inhibitor on downstream signaling of TNF-α, RhoA and Rac, in HUVECs. Effects of ROCK inhibitor on TNF-α-induced phosphorylation of MYPT (upper graph) and ERK1/2 (lower graph) were examined in HUVECs. After the pre-treatment with ROCK inhibitor (10 μM Y-27632) for 1 h, HUVECs were stimulated with TNF-α (50 ng/mL) in the presence or absence of S1PC (300 μM) for 10, 20, 40 or 60 min. Cell lysates were analyzed by western blotting with indicated antibodies. Quantitative data are shown as mean ± SD, n = 3. Significant difference compared to the control group (^##^*p* < 0.01, ^#^*p* < 0.05) or TNF-α-treated group with the same treatment time (**p < 0.01, *p < 0.05) was determined by Bonferroni’s multiple comparison test.

**Additional file 8: Figure S8.** Effects of MEK1 inhibitor on downstream signaling of TNF-α, RhoA and Rac, in HUVECs. Effects of MEK1 inhibitor on TNF-α-induced protein expression of MLCK (**a**) and MYPT phosphorylation (**b**) were examined in HUVECs. After the pre-treatment with MEK1 inhibitor (30 μM PD98059) for 1 h, HUVECs were stimulated with TNF-α (50 ng/mL) in the presence or absence of S1PC (300 μM) for 24 h (**a**) or indicated periods (**b**). Cell lysates were analyzed by western blotting with indicated antibodies. Quantitative data are shown as mean ± SD, n = 3. Significant difference compared to the control group (^##^*p* < 0.01, ^#^*p* < 0.05) or TNF-α-treated group with the same treatment time (**p < 0.01, *p < 0.05) was determined by Bonferroni’s multiple comparison test.

**Additional file 9: Figure S9.** Effects of ROCK and MEK1 inhibitors on TNF-α-induced hyperpermeability of HUVECs. After the pre-treatment with ROCK (10 μM Y-27632 (**a**)) or MEK1 inhibitor (30 μM PD98059 (**b**)) for 1 h, HUVECs were stimulated with TNF-α (50 ng/mL) in the presence or absence of S1PC (300 μM) for 24 h, and then vascular permeability assay was conducted. Data are shown as mean ± SD, n = 3–4. Significant difference compared to the control group (^##^*p* < 0.01, ^#^*p* < 0.05) or TNF-α-treated group (*p < 0.05) was determined by Bonferroni’s multiple comparison test. N.S. indicates no statistical significance (*p* > 0.05).

## Data Availability

The datasets used and/or analysed during the current study are available from the corresponding author on reasonable request.

## Abbreviations

AJ: Adherens junction
AGE: Aged garlic extract
BSA: Bovine serum albumin
DAPI: 4′,6-Diamidino-2-phenylindole
Elk1: ETS domain-containing transcription factor 1
GAPDH: Glyceraldehyde 3-phosphate dehydrogenase
GEF: Guanine nucleotide exchange factor
HRP: Horseradish peroxidase
HUVECs: Human umbilical vein endothelial cells
MLB: Mg^2^^+^ lysis/wash buffer
MLC: Myosin light chain
MLCK: MLC kinase
MLCP: MLC phosphatase
MYPT: MLCP regulatory subunit
PBS: Phosphate buffered saline
RIPA: Radioimmunoprecipitation assay
S1PC: *S*-1-Propenylcysteine
SAC: *S*-Allylcysteine
SAMC: *S*-Allylmercaptocysteine
SD: Standard deviation
siRNA: Small interfering RNA
TJ: Tight junction
TNF-α: Tumor necrosis factor-α
TNFR: TNF receptor
TRAF2: TNFR-associated factor 2
VE-cadherin: Vascular endothelial cadherin
ZO: Zona occludens
