# Supplementary figures and images for: S-1-propenylcysteine improves TNF-α-induced vascular endothelial barrier dysfunction by suppressing the GEF-H1/RhoA/Rac pathway
Source: Cell Commun Signal. 2021 Feb 15;19:17. doi: 10.1186/s12964-020-00692-w (PMC7883441; doi:10.1186/s12964-020-00692-w)

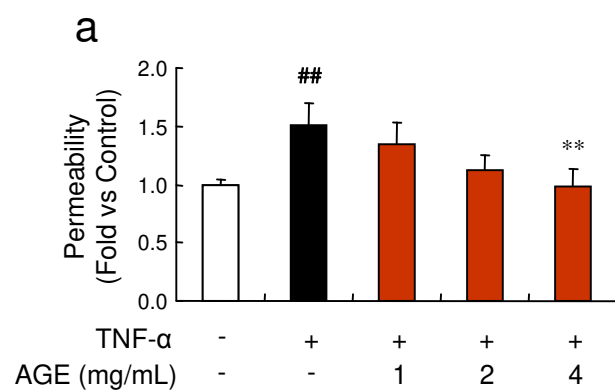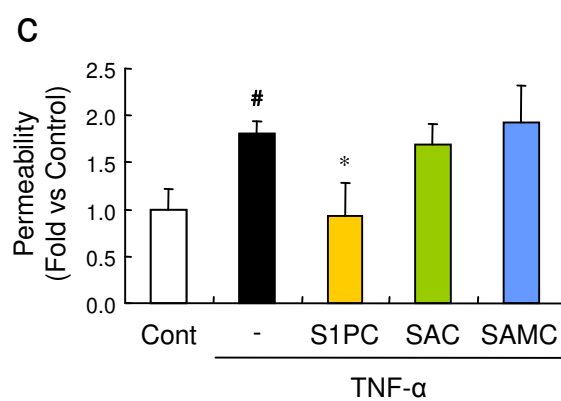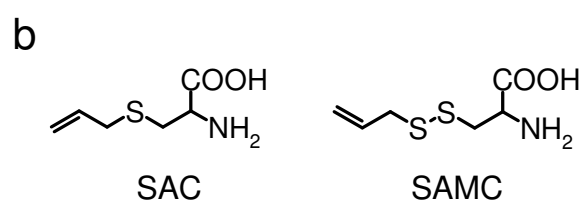

Supplement: Supplementary file 2 — Additional file 1: Figure S1. Effects of AGE and its major sulfur-constituents on TNF-α-induced hyperpermeability of HUVECs. a The concentration-dependent effect of AGE on TNF-α-induced hyperpermeability of HUVECs. Vascular permeability assay was conducted after the stimulation with TNF-α (50 ng/mL) in the presence or absence of AGE (1, 2 or 4 mg/mL) for 24 h. Data are shown as mean ± SD, n = 3–4. Significant difference compared to the control group (##p < 0.01) or TNF-α-treated group (**p < 0.01) was determined by Dunnett’s multiple comparison test. b Chemical structures of SAC and SAMC. c Effects of major sulfur-containing constituents of AGE on TNF-α-induced hyperpermeability of HUVECs. Vascular permeability was conducted after the stimulation with TNF-α (50 ng/mL) in the presence or absence of sulfur-containing constituents of AGE (300 μM S1PC, SAC or SAMC) for 24 h. Data are shown as mean ± SD, n = 4. Significant difference compared to the control group (#p < 0.05) or TNF-α-treated group (*p < 0.05) was determined by Bonferroni’s multiple comparison test. [file 12964_2020_692_MOESM2_ESM.pdf]

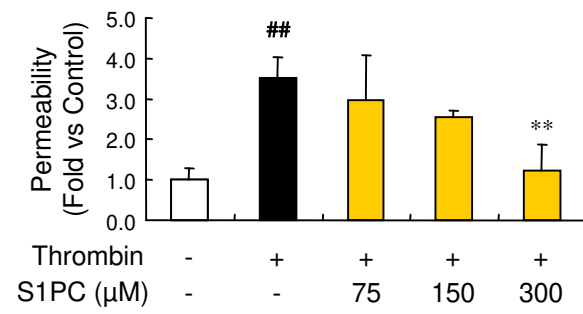

Supplement: Supplementary file 3 — Additional file 2: Figure S2. The concentration-dependent effect of S1PC on thrombin-induced hyperpermeability of HUVECs. Vascular permeability assay was conducted after the stimulation with thrombin (1 U/mL) in the presence or absence of S1PC (75, 150 or 300 μM) for 24 h. Data are shown as mean ± SD, n = 4. Significant difference compared to the control group (##p < 0.01) or TNF-α-treated group (**p < 0.01) was determined by Dunnett’s multiple comparison test. [file 12964_2020_692_MOESM3_ESM.pdf]

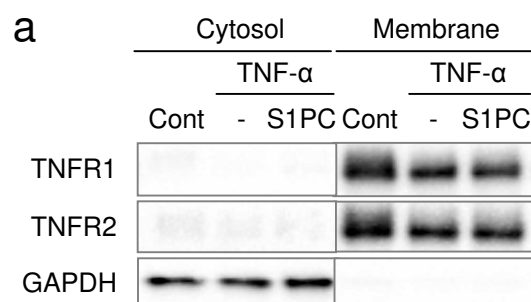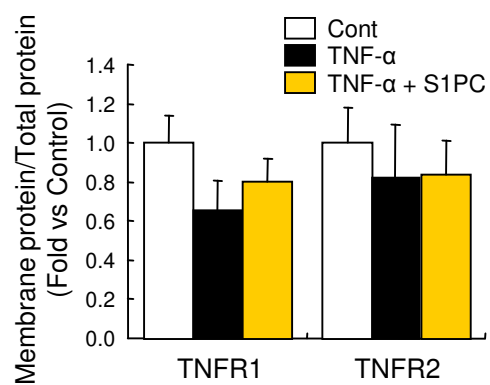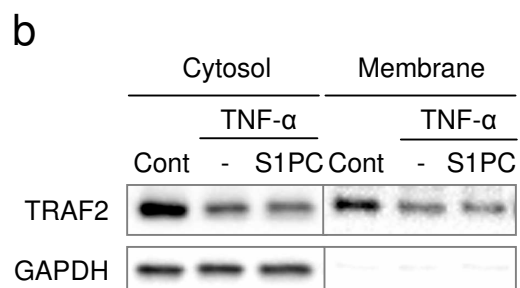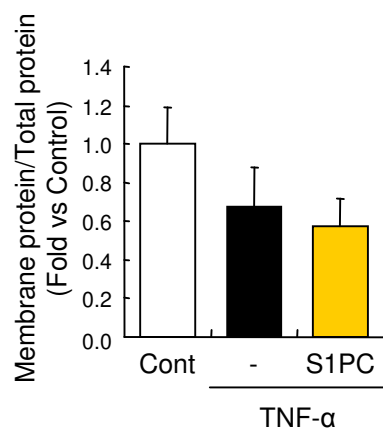

Supplement: Supplementary file 4 — Additional file 3: Figure S3. Effects of S1PC on protein levels of TNF receptors and TRAF2 in HUVECs. a Effects of S1PC on the protein levels of TNFR1 and TNFR2 in HUVECs. The membrane and cytoplasmic proteins were extracted after the stimulation with TNF-α (50 ng/mL) in the presence or absence of S1PC (300 μM) for 24 h and analyzed by western blotting with indicated antibodies. Quantitative data are shown as mean ± SD, n = 3. b Effect of S1PC on the protein level of adaptor protein TRAF2 in HUVECs. The membrane and cytoplasmic proteins were extracted after the stimulation with TNF-α (50 ng/mL) in the presence or absence of S1PC (300 μM) for 30 min and analyzed by western blotting with indicated antibodies. Quantitative data are shown as mean ± SD, n = 4. [file 12964_2020_692_MOESM4_ESM.pdf]

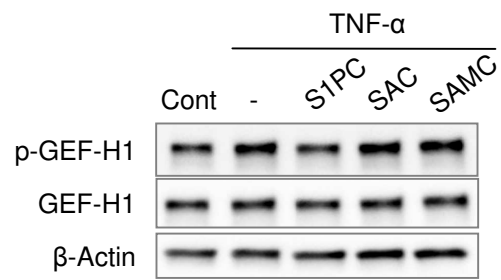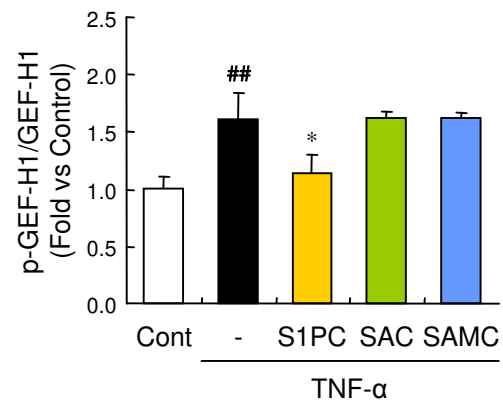

Supplement: Supplementary file 5 — Additional file 4: Figure S4. Effects of major sulfur-containing constituents of AGE on TNF-α-induced GEF-H1 phosphorylation in HUVECs. Cell lysates were obtained after the stimulation with TNF-α (50 ng/mL) in the presence or absence of sulfur-containing constituents of AGE (300 μM S1PC, SAC and SAMC) for 30 min and analyzed by western blotting with indicated antibodies. Quantitative data are shown as mean ± SD, n = 3. Significant difference compared to the control group (##p < 0.01) or TNF-α-treated group (*p < 0.05) was determined by Bonferroni’s multiple comparison test. [file 12964_2020_692_MOESM5_ESM.pdf]

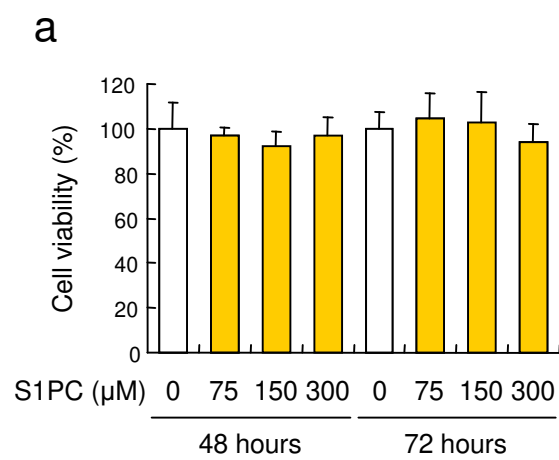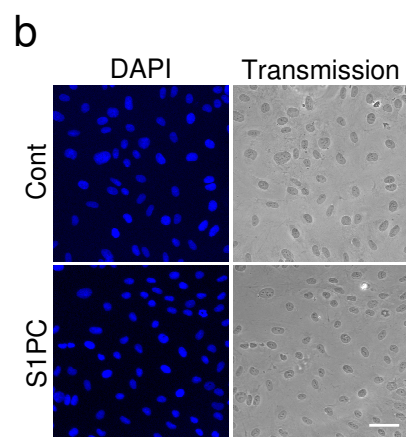

Supplement: Supplementary file 6 — Additional file 5: Figure S5. Effects of S1PC on the cell proliferation and morphology of HUVECs. a Effect of S1PC on the cell proliferation in HUVECs. Cell viability was measured with cell proliferation assay system after the treatment with S1PC (75, 150 or 300 μM) for 48 and 72 h. Data are shown as mean ± SD, n = 3. b Effect of S1PC on the cell morphology in HUVECs. Cell nuclei were stained with DAPI to assess normal cytokinesis after the treatment with S1PC (300 μM) for 24 h. Specific fluorescence: blue for nuclei stained with DAPI. Scale bar, 100 μm. [file 12964_2020_692_MOESM6_ESM.pdf]

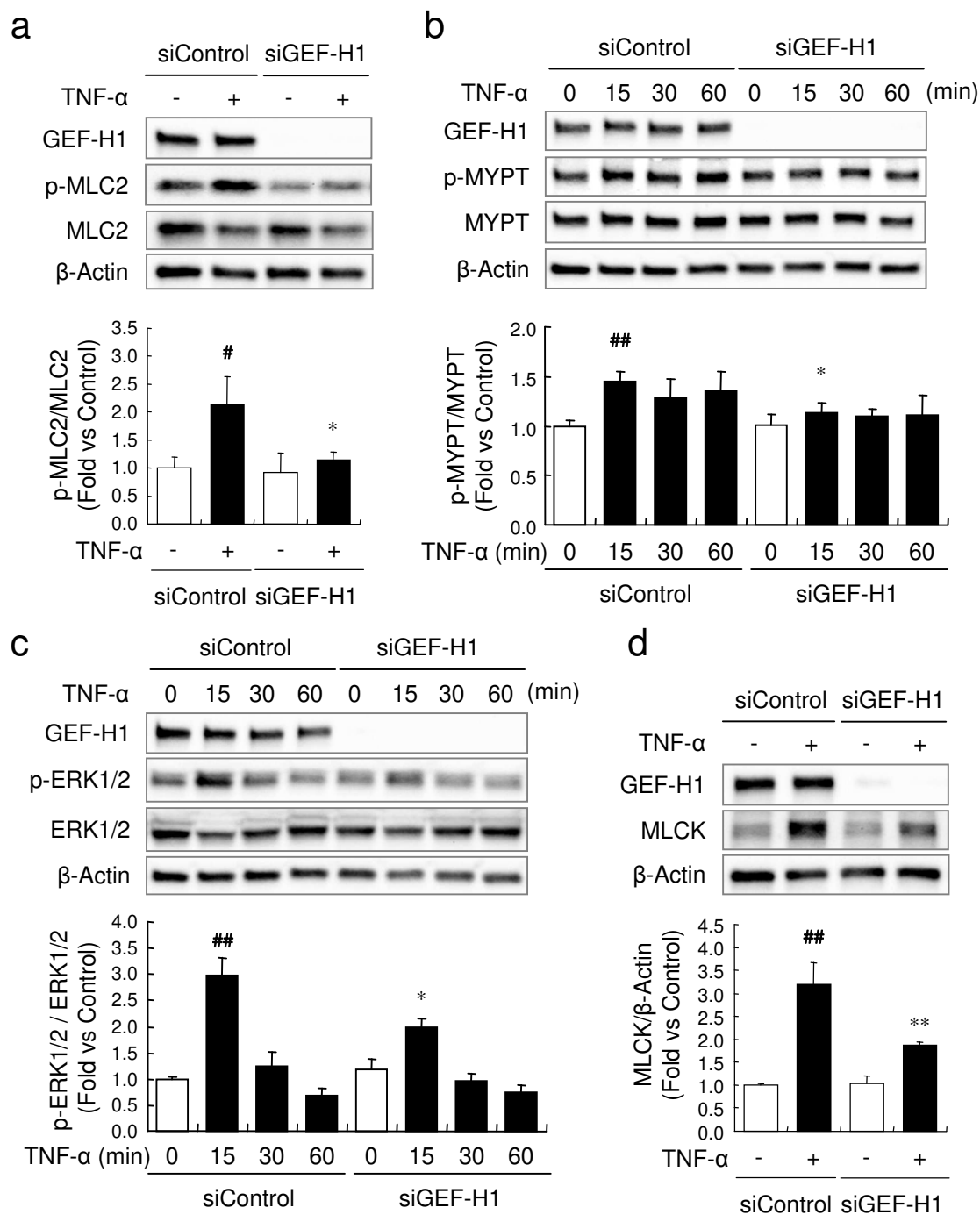

Supplement: Supplementary file 7 — Additional file 6: Figure S6. Effects of GEF-H1 knockdown on downstream signaling of TNF-α, RhoA and Rac, in HUVECs. Effects of GEF-H1 knockdown on TNF-α-induced MLC2 phosphorylation (a), MYPT phosphorylation (b), ERK1/2 phosphorylation (c) and MLCK protein expression (d) were examined in HUVECs. HUVECs were transfected with control siRNA (siControl) or GEF-H1-targeting siRNA (siGEF-H1). Cell lysates were obtained after the stimulation with TNF-α (50 ng/mL) for 24 h (a, d) or indicated periods (b, c), and analyzed by western blotting with indicated antibodies. Quantitative data are shown as mean ± SD, n = 3. Significant difference compared to non-treatment siControl group (##p < 0.01, #p < 0.05) or TNF-α-treated siControl group with the same treatment time (**p < 0.01, *p < 0.05) was determined by Bonferroni’s multiple comparison test. [file 12964_2020_692_MOESM7_ESM.pdf]

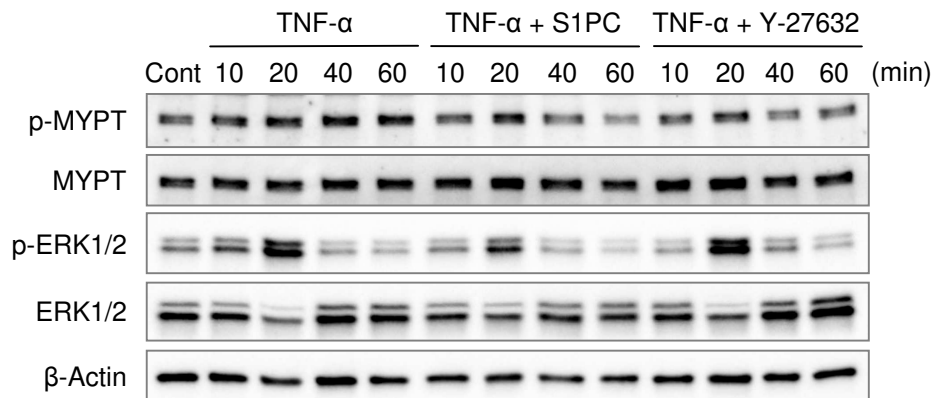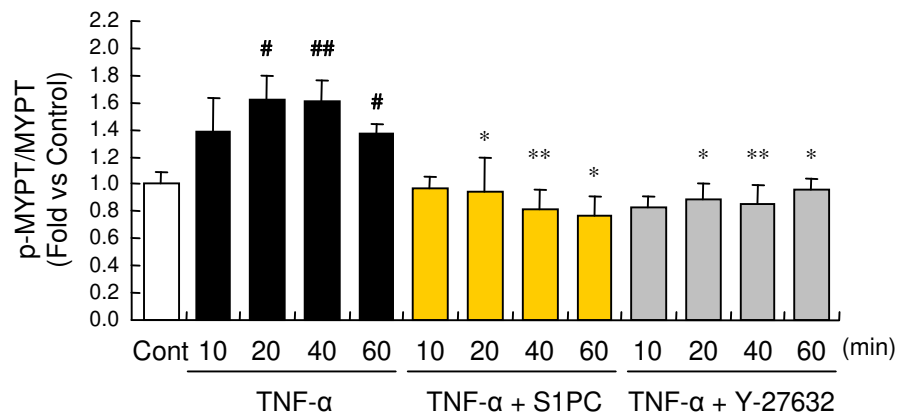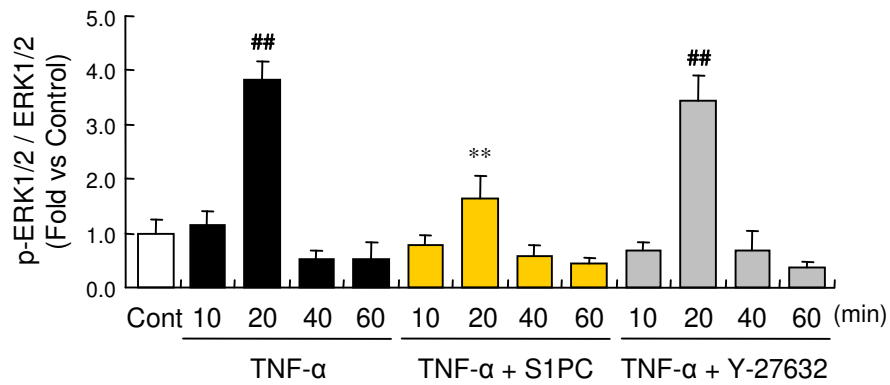

Supplement: Supplementary file 8 — Additional file 7: Figure S7. Effects of ROCK inhibitor on downstream signaling of TNF-α, RhoA and Rac, in HUVECs. Effects of ROCK inhibitor on TNF-α-induced phosphorylation of MYPT (upper graph) and ERK1/2 (lower graph) were examined in HUVECs. After the pre-treatment with ROCK inhibitor (10 μM Y-27632) for 1 h, HUVECs were stimulated with TNF-α (50 ng/mL) in the presence or absence of S1PC (300 μM) for 10, 20, 40 or 60 min. Cell lysates were analyzed by western blotting with indicated antibodies. Quantitative data are shown as mean ± SD, n = 3. Significant difference compared to the control group (##p < 0.01, #p < 0.05) or TNF-α-treated group with the same treatment time (**p < 0.01, *p < 0.05) was determined by Bonferroni’s multiple comparison test. [file 12964_2020_692_MOESM8_ESM.pdf]

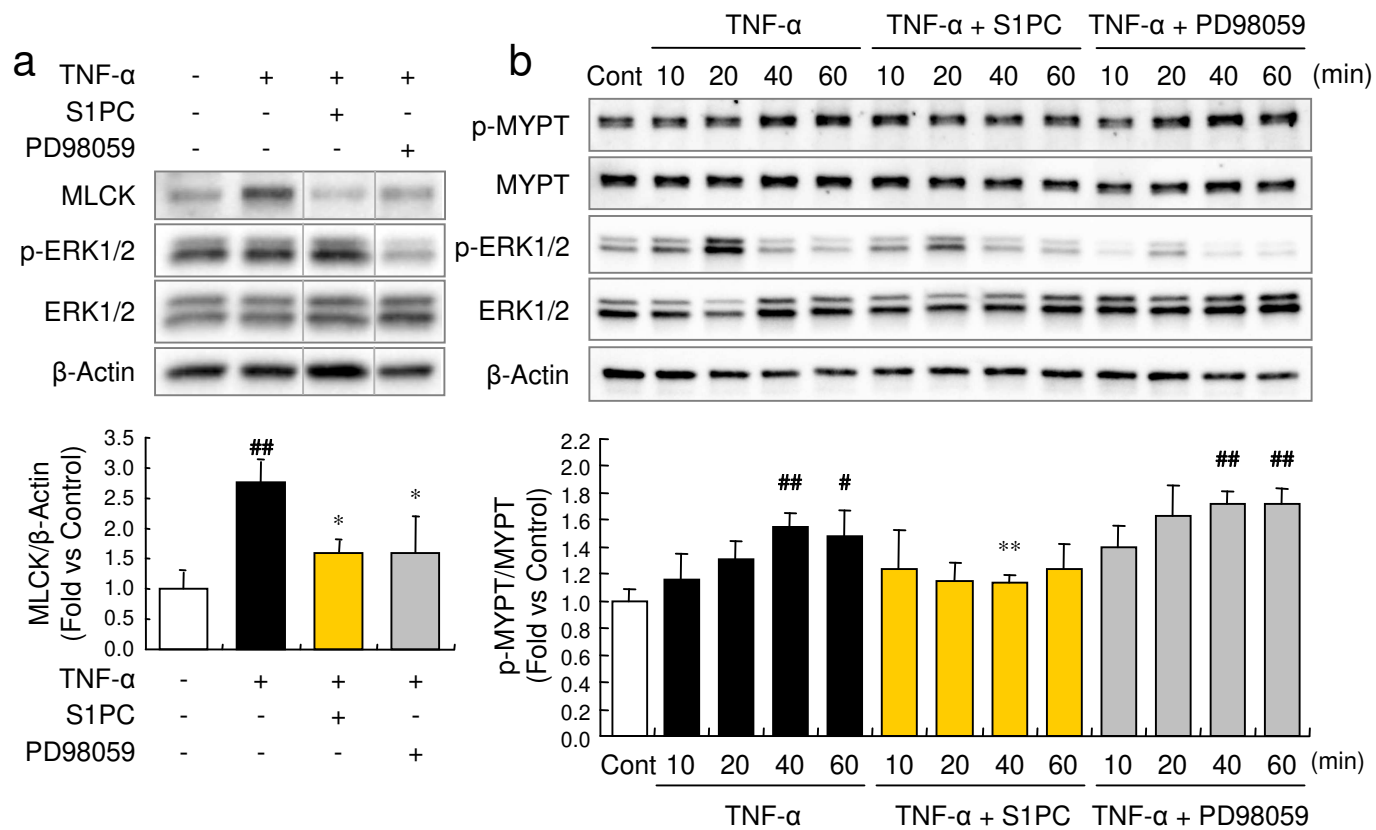

Supplement: Supplementary file 9 — Additional file 8: Figure S8. Effects of MEK1 inhibitor on downstream signaling of TNF-α, RhoA and Rac, in HUVECs. Effects of MEK1 inhibitor on TNF-α-induced protein expression of MLCK (a) and MYPT phosphorylation (b) were examined in HUVECs. After the pre-treatment with MEK1 inhibitor (30 μM PD98059) for 1 h, HUVECs were stimulated with TNF-α (50 ng/mL) in the presence or absence of S1PC (300 μM) for 24 h (a) or indicated periods (b). Cell lysates were analyzed by western blotting with indicated antibodies. Quantitative data are shown as mean ± SD, n = 3. Significant difference compared to the control group (##p < 0.01, #p < 0.05) or TNF-α-treated group with the same treatment time (**p < 0.01, *p < 0.05) was determined by Bonferroni’s multiple comparison test. [file 12964_2020_692_MOESM9_ESM.pdf]

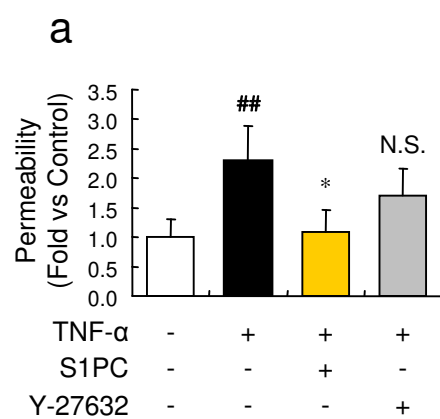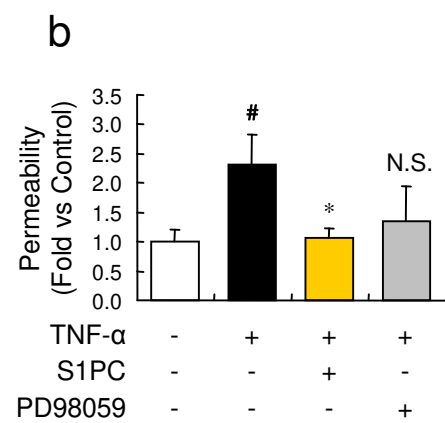

Supplement: Supplementary file 10 — Additional file 9: Figure S9. Effects of ROCK and MEK1 inhibitors on TNF-α-induced hyperpermeability of HUVECs. After the pre-treatment with ROCK (10 μM Y-27632 (a)) or MEK1 inhibitor (30 μM PD98059 (b)) for 1 h, HUVECs were stimulated with TNF-α (50 ng/mL) in the presence or absence of S1PC (300 μM) for 24 h, and then vascular permeability assay was conducted. Data are shown as mean ± SD, n = 3–4. Significant difference compared to the control group (##p < 0.01, #p < 0.05) or TNF-α-treated group (*p < 0.05) was determined by Bonferroni’s multiple comparison test. N.S. indicates no statistical significance (p > 0.05). [file 12964_2020_692_MOESM10_ESM.pdf]
